# Gestational glucose intolerance among pregnant women at the Cape Coast Teaching Hospital

**DOI:** 10.1186/s12884-024-06568-y

**Published:** 2024-05-14

**Authors:** Nelson Ekow Kumah, Evans Kofi Agbeno, Leonard Derkyi-Kwarteng, Ebenezer Aniakwaa-Bonsu, Sampson Kafui Djonor, Samuel Acquah

**Affiliations:** 1 Department of Microbiology and Immunology, School of Medical Sciences, College of Health and Allied Sciences, University of Cape Coast, Cape Coast, Ghana; 2 Department of Obstetrics and Gynaecology, School of Medical Sciences, College of Health and Allied Sciences, University of Cape Coast, Cape Coast, Ghana; 3 Department of Pathology, School of Medical Sciences, College of Health and Allied Sciences, University of Cape Coast, Cape Coast, Ghana; 4 Department of Epidemiology and Disease Control, School of Public Health, University of Ghana, Accra, Ghana; 5 Department of Medical Biochemistry, School of Medical Sciences, College of Health and Allied Sciences, University of Cape Coast, Cape Coast, Ghana

**Keywords:** Pregnancy, Insulin resistance, Gestational glucose intolerance, Gestational diabetes mellitus, Malaria

## Abstract

**Background:**

Malaria in pregnancy can have adverse outcomes if untreated. Both malaria and pregnancy are associated with insulin resistance and diabetes. Although malaria is treated prophylactically with gestational diabetes mellitus (GDM) screened for in pregnancy as part a routine antenatal care, their impacts have not been examined in terms of other forms of dysglycaemia. This cross-sectional study examined insulin resistance and its relationship with dysglycaemia and malaria among pregnant women in the Cape Coast Teaching Hospital (CCTH).

**Methods:**

Using a structured questionnaire, demographic and clinical information were obtained from 252 pregnant women aged 18–42 years. Weight and height were measured for computation of body mass index (BMI). Measurement of insulin, lipid profile and glucose were taken under fasting conditions followed by oral glucose tolerant test. Insulin resistance and beta-cell function were assessed by the homeostatic model as malaria was diagnosed by microscopy.

**Results:**

The respective prevalence of GDM, gestational glucose intolerance (GGI) and insulin resistance were 0.8% (2/252), 19.44% (49/252) and 56.75% (143/252). No malaria parasite or dyslipidaemia was detected in any of the participants. Apart from BMI that increased across trimesters, no other measured parameter differed among the participants. Junior High School (JHS) education compared with no formal education increased the odds (AOR: 2.53; CI: 1.12–5.71; *P* = 0.03) but 2^nd^ trimester of pregnancy compared to the 1^st^ decreased the odds (AOR: 0.32; CI: 0.12–0.81; *P* = 0.02) of having insulin resistance in the entire sample. In a sub-group analysis across trimesters, pregnant women with JHS education in their 3^rd^ trimester had increased odds (AOR: 4.41; CI: 1.25–15.62; *P* = 0.02) of having insulin resistance.

**Conclusion:**

Prevalence of GDM and GGI were 0.8% and 19.44% respectively. The odds of insulin resistance increased in pregnant women with JHS education in the 3^rd^ trimester. Appropriate measures are needed to assuage the diabetogenic risk posed by GGI in our setting.

**Supplementary Information:**

The online version contains supplementary material available at 10.1186/s12884-024-06568-y.

## Introduction

According to the International Diabetes Federation (IDF), the prevalence of diabetes keeps rising globally with 537 million people with the condition in 2021 expected to rise to 783 million by 2045 [1]. The African region is expected to experience the highest increase in incidence of 129% suggesting that the 24 million people aged 20–79 years with the condition will rise to 55 million by 2045. Moreover, the region continues to bear the highest prevalence (53.6%) of undiagnosed cases of the condition globally. Insulin resistance (IR), an independent risk factor for the development of type 2 diabetes mellitus (T2DM) and other metabolic syndromes, is a pathological state characterized by failure of the insulin system [2]. It is a decreased sensitivity of insulin-sensitive cells to actions mediated by insulin. With T2DM constituting over 90% of global diabetes cases [1], investigating factors that can contribute to the development of IR, which predates the development of full-blown T2DM, is critical in our quest to minimise impact of the bleak diabetogenic risk predictions in the African region. Pregnancy is a naturally critical period in a woman’s life, which comes with adaptation and probable health risk to the woman and foetus. Although pregnancy is known to cause IR state, IR has been associated with gestational diabetes mellitus (GDM) [3, 4]. GDM predisposes the affected to future development of T2DM [1, 5] and is affected by several other factors including smoking, history of GDM, still birth or infants with congenital abnormality, overweight/obesity, excessive weight gain during pregnancy, polycystic ovarian syndrome and older age [1].

Malaria is another condition that has been associated with IR in adult and children [6, 7]. It is a condition that can be considered an African problem since the continent continues to bear over 90% of the global burden of malaria and its associated deaths [8]. Ghana as a malaria-endemic country has its entire population at risk of the condition. In spite of tremendous progress made at reducing the disease burden, it continues to be the leading cause of morbidity, responsible for 38% of all outpatient visits to health facilities in the country in recent times [9], with pregnant women and children under five years being the most affected [8]. *Plasmodium falciparum* species accounts for more than 97% of the global malaria burden. In recognition of the probable adverse effects of malaria in pregnancy [10–12], especially, the asymptomatic [13, 14] form, prophylactic treatment with sulfadoxine–pyrimethamine is administered to eligible pregnant women in line with the updated recommendation of the World Health Organization (WHO) on intermittent preventive treatment in pregnancy [15]. Although IR has been associated with malaria [6, 7] and GDM [3, 4] in separate studies, scientific information on the relationship among IR, malaria and dysglycaemia in pregnancy in a single study is limited in our setting. Therefore, the current study was designed to examine the relationship among IR, malaria and dysglycaemia in pregnant women receiving antenatal care at the Cape Coast Teaching Hospital (CCTH) of Ghana. In our context, dysglycaemia refers to GDM and other forms of hyperglycaemia such as gestational glucose intolerance (GGI). GGI refers to hyperglycaemia below the threshold for diagnosing GDM but above the normal level after a standard glucose load.

## Materials and methods

### Study area

The Obstetrics and Gynaecology Department of the Cape Coast Teaching Hospital (CCTH) served as the study site. Being a tertiary healthcare facility, the CCTH serves as a referral hospital for various health facilities in the Central and Western regions of Ghana. The region occupies a total area of 9,826 km^2^ with Cape Coast as its capital. The population of the region was 2,859,821 as of the 2021 National Population and Housing Census [16], with 189,925 living in the Cape Coast city. The Obstetrics and Gynaecology Department of the CCTH provides comprehensive antenatal care to clients who visit the facility for various services.

### Study design, sample size estimation and selection of participants

A cross-sectional study design was employed in this study at the CCTH. Participants were selected from a pool of pregnant women who visited the CCTH for various antenatal care. The sample size for the study was calculated using the formula described elsewhere [17], which relies on prevalence rate. Using gestational prevalence rate of 14.28% for sub-Saharan Africa [18], the sample size was estimated as;

$\text{N}=\frac{{\text{z}}^{2}\times (\text{p}\times \text{q})}{{\text{d}}^{2}}$ Where; N = sample size;

z = the critical probability value for a confidence level of 95% (1.96),

p = estimated proportion of gestational diabetes of 14.28% [18];

q = 1-p, and d = margin of error (0.05);

$$\text{N}=\frac{{1.96}^{2}\times (0.1428\times 0.8572)}{{0.05}^{2}} = \frac{3.8416\times 0.1224}{0.0025}= \frac{0.4702432}{0.0025}= 188$$

**Fig. 1:**
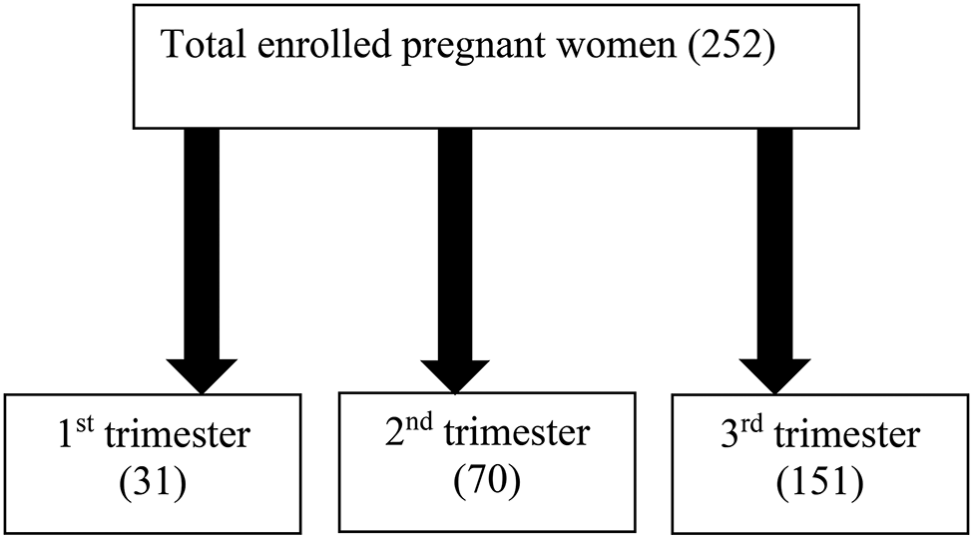
Number of women per trimester of pregnancy

Assuming a 20% (*n* = 38) nonresponse rate, the study was to involve a minimum of 226 participants. The study, however, involved 252 pregnant women who presented at the antenatal clinic (ANC) of the CCTH during the study period. Pregnant women aged 18 years and above who were apparently healthy and willing to participate were included in the study. However, pregnant women diagnosed with diabetes, hepatitis, human immunodeficiency virus or any pregnancy-associated health condition known to affect any of the measured indices were excluded from the study. Using the systematic random sampling technique, every third pregnant woman who visited the ANC and satisfied the inclusion criteria was selected after the first person was recruited for the study. Recruitment of participants started from 2^nd^ June, 2022 and ended on 27^th^ July, 2023. Participants were selected across the various trimesters of pregnancy (Fig. 1).

### Sociodemographic information, anthropometry and laboratory measurements

#### Sociodemographic information

A questionnaire was designed (supplementary file) for the collection of relevant sociodemographic and clinical information including age, marital status, use of insecticide treated mosquito net, and other medical and obstetric information of study participants.

#### Body mass index estimation

Height and weight were measured to the nearest 0.1 cm and 0.1 kg respectively for computation of body mass index (BMI). Height and weight were measured with a stadiometer and electronic weighing scale respectively. BMI was calculated as the ratio of weight in kilogrammes (kg) to the square of the height in metres (m).

#### Estimation of fasting plasma glucose and oral glucose tolerance test

After 8-12-hour overnight fast, 2 ml venous blood (mid-cubital) sample was taken from each participant into a sodium fluoride tube for preparation of plasma following standard procedures at CCTH. Plasma glucose was measured within 30 min of sampling in a routine manner using the Selectra Pro XL biochemistry analyzer (Elitech Group, Netherlands) following the manufacturer’s instructions.

Oral glucose tolerance test (OGTT) was performed according to the WHO criteria [19]. OGTT was performed by oral administration of 75 g glucose dissolved in 250 ml of water to each participant, followed by hourly measurements of plasma glucose with a glucometer (OneTouch Select Plus, Lifescan Inc., USA) for three hours in accordance with the routine procedure at CCTH. Glucose measurements by the glucometer have been duly validated against those of the autonalyzer and found to be comparable in the routine procedure at CCTH. Glycaemia classification was based on the World Health Organization (WHO) diagnostic criteria of 2013 [20]. Participants with fasting plasma glucose (FPG) < 5.1 mmol/l or 1-hr OGTT < 10 mmol/l or 2-hr OGTT < 8.6 mmol/l were considered to be normal. Those with FPG ≥ 5.1 mmol /l, and / or 1-hr OGTT ≥ 10 mmol/l, and / or 2-hr OGTT ≥ 8.6 mmol/l were considered to have gestational diabetes. However, participants with FPG ≥ 7 mmol/l and/or 2-hr- OGTT ≥ 11.1 mmol/L irrespective of pregnancy trimester were diagnosed with diabetes in pregnancy. Gestational glucose intolerance (GGI) was diagnosed for participants with 2-hr OGTT ≥ 6.7 mmol/l but ≤ 7.7 mmol/l [21].

#### Measurement of lipid profile

Lipid profile was assessed in a routine manner with the automated Agappe Pro CLX Chemistry Analyzer. Dyslipidaemias were defined as: total cholesterol (TC) ≥ 5.2 mmol/l, triglyceride (TG) ≥ 1.69 mmol/l, low-density lipoprotein cholesterol (LDL) ≥ 3.4 mmol/l and high-density lipoprotein cholesterol (HDL) < 1.29 mmol/l.

#### Estimation of plasma insulin

Insulin was measured using a solid-phase sandwich enzyme-linked immuno-sorbent assay (ELISA) technique. The assay relies on immobilization of an anti-insulin antibody on a solid phase (microtiter wells) and a second anti-insulin antibody in the antibody-enzyme conjugate solution. Upon addition of the appropriate specimen, insulin is sandwiched between the two antibodies. The absorption of light by the insulin-antibody complex in solution is proportional to the amount of insulin present in the specimen which can then be measured spectrophotometrically. Specifically, after reagents and samples were brought to room temperature, 50 𝜇l of various concentrations of insulin standard (0, 5, 25, 50, 100 and 200 𝜇IU/ml) were pipetted into microplate wells, followed by an equal volume of the sample. Exactly 100 𝜇l of 1X enzyme conjugate was then added to each well, mixed gently for 30 s and incubated for one hour at room temperature in accordance with manufacturer’s (PerkinELmer Health Sciences Inc., USA) instructions. Using an automated microplate washer (Thermo Electron Co-Operation, Finland), the microplate wells were then washed 5 times each with 300 𝜇l of 1X wash buffer. Exactly 100 𝜇l of TMB substrate was then added to each well followed by incubation at room temperature for 20 min in the dark. The reaction was then stopped by the addition of 100 𝜇l stop solution, mixed gently for 10 s until the blue colouration changed to yellow. Absorbance was then read within 15 min at 450 nm using a multiscan microplate reader (Thermo-Scientific, Finland). Appropriate standard curve was prepared from absorbance values of the insulin standards. The concentration of insulin in sample was subsequently determined from the standard curve.

#### Determination of beta-cell function and insulin resistance

The homeostatic model for assessment of beta-cell function, HOMA - B = 20 * Fasting Insulin $({{{\rm{mU}}} \over 1})/$ Fasting Glucose $({{{\rm{mmol}}} \over 1})\; - \;3.5$, and insulin resistance, HOMA - IR = Glucose $({{{\rm{mmol}}} \over 1})\; - \;3.5$ * Insulin $({{{\rm{mU}}} \over 1})/22.5$ developed by Mathews et al. [22] was applied in the assessment of beta-cell function and insulin resistance in the study participants. Insulin resistance was defined as HOMA-IR > 2.6 [23].

#### Diagnosis of malaria

Malaria was diagnosed by microscopy by an experienced microscopist at the CCTH. Microscopy remains the gold standard for malaria diagnosis. Thick film was used for parasite count as thin film was reserved for species identification. A well-mixed 6 µl and 10 µl blood were placed on a clean glass slide for the thin and thick films respectively, followed by Giemsa staining, air-dried and observed under oil immersion at 100X in line with standard procedures at CCTH. The parasite density was calculated using the formula,

$${\rm{Parasite}}\;{\rm{density}}\left( {{\rm{per}}\;{\rm{\mu l}}} \right) = {{{\rm{number}}\;{\rm{of}}\;{\rm{parasites}}\;{\rm{counted}}\; \times \;8000} \over {{\rm{Number}}\;{\rm{of}}\;{\rm{leukocytes}}\;{\rm{counted}}}}$$

A blood film was considered negative when examination of 1000 white blood cells reveal no asexual parasites.

### Ethical considerations

Prior to the commencement of the study, ethical clearance was sought and granted by the Cape Coast Teaching Hospital Ethical Review Committee (CCTHERC/EC/2022/089). In addition, a written informed consent was obtained from each participant. All protocols employed were in strict adherence to the ethical standards of CCTH, Ghana Health Service and the World Medical Association Declaration of Helsinki.

### Statistical analysis

Statistical Software for Data Science (STATA) version 15 Corp (StataCorp LLC, USA) software was used for data analyses. Categorical variables are presented in percentages with continuous variables presented as mean ± standard deviation (SD) if normally distributed or median and inter-quartile range (IQR) where appropriate. Mean and median values across trimesters were compared with one-way analysis of variance (ANOVA) and the Kruskal-Wallis test respectively, followed by appropriate post-hoc tests. Multivariate logistic regression analysis was applied to identify predictors of insulin resistance irrespective of pregnancy duration in the entire sample and across trimesters after controlling for appropriate confounders including marital status, educational level, birthweight of last pregnancy and lipid profile, where appropriate. Crude and adjusted odds ratios were computed with 95% confidence intervals. A p-value < 0.05 was considered statistically significant for all analyses.

## Results

The study involved 252 apparently healthy pregnant women aged 18–42 years. A 0.8% (2/252) and 19.44% (49/252) prevalence of GDM and GGI respectively were found in the current study. In terms of trimester, GGI prevalence of 9.68% (3/31), 18.57% (13/70), and 21.85% (33/151) was observed for 1^st^, 2^nd^ and 3^rd^ trimester respectively. Prevalence of insulin resistance was 56.75% (143/252) in the study participants.

In Table 1, the demographic and clinical characteristics of participants are shown. Of the 252 pregnant women who participated in the study, most were married 74.21% (187/252), without formal education 47.62% (120/252), normotensive 96.43% (243/252), did not take alcohol 95.24% (240/252), were in the 3^rd^ trimester 59.92% (151/252) and had normal birthweight of last pregnancy 91.67 (231/252). The mean age and BMI were 30 years and 30.48 kg/m^2^ respectively (Table 1).

**Table 1:** Demographic and clinical characteristics of participants

Parameter	Number (%) or mean ± standard deviation
Age	30.12 ± 5.53
BMI	30.48 ± 4.51
**Marital status**	
Divorced	23(9.13)
Married	187(74.21)
Single	42(16.67)
**Education**	
Tertiary	24(9.52)
Elementary	9(3.57)
JHS	42(16.67)
SHS	57(22.62)
No formal education	120(47.62)
**Trimester**	
1	31(12.30)
2	70(27.78)
3	151(59.92)
**Birthweight of last pregnancy**	
Normal	231(91.67)
Underweight	8(3.17)
Overweight	13(5.16)
**Hypertension**	
No hypertension	243(96.43)
Hypertension	9(3.576)
**Alcohol consumption**	
No	240(95.24)
Yes	4.76)

A comparison of the various measured indices across trimesters, did not show any statistically (*P* > 0.05; Table 2) significant difference in level of any of the parameters except BMI which differed significantly (*P* < 0.001; Table 2) across trimesters, and increased along the gestation period.

**Table 2:** Levels of measured indices across trimesters

Parameter	All	1st Trimester	2nd Trimester	3rd Trimester	*p*-value
FPG (mmol/l)	4.70(4.21–5.10)	4.60(4.20-5.00)	4.60(4.20-5.00)	4.70(4.21–5.10)	0.71
LDL (mmol/l)	2.65(1.90–3.44)	2.50(1.91–3.90)	2.74(1.80–3.62)	2.61(1.90–3.40)	0.93
TC (mmol/l)	4.53(3.85–5.34)	4.31(3.52–5.76)	4.59(3.84–5.76)	4.58(3.85–5.34)	0.62
HDL (mmol/l)	1.32 ± 0.34	1.19 ± 0.36	1.32 ± 0.30	1.34 ± 0.36	0.07
TG (mmol/l)	1.26(0.95–1.54)	1.40(0.98–1.85)	1.34(0.92–1.64)	1.24(0.95–1.54)	0.43
BMI (kg/m^2^)	30.48 ± 4.51	25.90 ± 2.34	29.07 ± 3.72	32.07 ± 4.34	< 0.001*
Insulin mIU/l	15.50(5-39.47)	24.79(5-40.53)	10.50(5-35.54)	15.52(5-40.5)	0.082
HOMA-IR (mIU/l)	3.32(1.07–7.73)	3.98(1.04–9.33)	2.10(1.02–6.68)	3.64(1.09–7.75)	0.20
HOMA-B (%)	56.37(23.56-173.73)	75.00(30.33-163.21)	33.59(17.33-170.25)	70.66(25.41-184.27)	0.09
OGTT1 (mmol/l)	6.97 ± 0.99	6.83 ± 0.98	6.86 ± 0.98	7.05 ± 1.00	0.31
OGTT2 (mmol/l)	6.09 ± 0.81	5.96 ± 0.87	5.97 ± 0.75	6.17 ± 0.81	0.14
OGTT3 (mmol/l)	4.96 ± 0.69	4.95 ± 0.89	4.91 ± 0.68	4.98 ± 0.66	0.79

*: *P* < 0.001 (including the least significant difference, LSD, post-hoc test); FPS: fasting plasma glucose; LDL: low-density lipoprotein cholesterol; HDL: high-density lipoprotein cholesterol; TC: total cholesterol; TG: triglycerides; BMI: body mass index; HOMA-IR: homeostatic model assessment of insulin resistance; HOMA-B: homeostatic model assessment of beta-cell function; OGTT: oral glucose tolerance test

In subsequent logistic regression analyses to identify predictors of insulin resistance among participants, having JHS education increased the odds (AOR: 2.53; CI: 1.12–5.71; *P* = 0.03; Table 3) but being in the 2^nd^ trimester decreased the odds of having insulin resistance (AOR: 0.32; CI: 0.12–0.81; *P* = 0.02; Table 3).

**Table 3:** Predictors of insulin resistance among participants

Parameter	No insulin resistance	Insulin resistant	COR (95%CI)	*p*-value	AOR (95%CI)	*p*-value
Marital status						
Single **(ref.)**	17(15.60)	25(17(0.48)	1		1	
Divorced	9(8.26)	14(9.79)	1.06(0.37–2.99)	0.92	0.79(0.24–2.67)	0.71
Married	83(76.15)	104(72.73)	0.81(0.33–1.95)	0.63	0.72(0.34–1.55)	0.40
**Formal education**						
No formal education **(ref.)**	60(55.05)	60(41.96)	1		1	
Primary	4(3.67)	5(3.50)	1.25(0.32–4.88)	0.75	0.63(0.14–2.79)	0.54
JHS	12(11.01)	30(20.98)	2.50(1.17–5.34)	0.02	2.53(1.12–5.71)	0.03
SHS	25(22.94)	32(22.38)	1.28(0.68–2.41)	0.45	1.23(0.62–2.43)	0.55
Tertiary	8(7.34)	16(11.19)	2.00(0.80–5.02)	0.14	1.59(0.58–4.35)	0.37
**Birthweight of last pregnancy**						
Normal	105(96.33)	126(88.11)	1			
Overweight	2(1.83)	11(7.69)	4.58(0.99–21.14)	0.05	3.44(0.71–1.69)6	0.13
Underweight	2(1.83)	6(4.20)	2.50(0.49–12.65)	0.27	2.56(0.48–13.58)	0.27
**Trimester**						
1	11(10.09)	20(13.99)	1		1	
2	41(37.61)	29(20.28)	0.39(0.16–0.93)	0.04	0.32(0.12–0.81)	0.02
3	57(52.29)	94(65.73)	0.91(0.41–2.03)	0.81	0.67(0.26–1.70)	0.40
**Total cholesterol**						
Normal	75(68.81)	94(65.73)	1		1	
High	34(31.19)	49(34.27)	1.15(0.68–1.96)	0.61	1.24(0.51–3.06)	0.33
**Triglyceride**						
Normal	84(77.06)	116(81.12)	1		1	
High	25(22.94)	27(18.88)	0.78(0.42–1.44)	0.43	0.40(0.16 − 0.10)	0.05
**HDL**						
Normal	89(81.65)	109(76.22)	1			
Low	20(18.35)	34(23.78)	1.39(0.74–2.57)	0.30	1.38(0.56–3.37)	0.49
**LDL**						
Normal	53(48.62)	70(48.95)	1		1	
High	56(51.38)	73(51.05)	0.99(0.60–1.62)	0.96	0.99(0.49–1.99)	0.97

HDL: high-density lipoprotein cholesterol; LDL: low-density lipoprotein cholesterol; JHS: junior high school; SHS: senior high school

In a sub-group analysis across trimesters, the odds of having insulin resistance was 4.41 times higher only in participants with JHS education who were in the 3^rd^ trimester (AOR: 4.41; CI: 1.25–15.62; *P* = 0.02; Table 4) of pregnancy compared to those without any formal education in the same trimester, after adjusting for appropriate confounders.

**Table 4:** Predictors of insulin resistance across trimesters of pregnancy

Parameter	Trimester 1 AOR (95% CI)	*P*-value	Trimester 2 AOR (95%CI)	*P*-value	Trimester 3 AOR (95% CI)	*P*-value
Marital status						
Single **(ref.)**	1		1		1	
Divorced	-		0.20(0.01–3.01)	0.25	0.73(0.14–3.77)	0.71
Married	5.71(0.14–23.46)	0.36	0.52(0.13–2.09)		0.59(0.20–1.79)	0.36
**Education**						
No formal education **(ref.)**	-	-	1		1	
Primary			1.18(0.04–31.53)	0.92	0.38(0.05–323)	0.38
JHS	2.83(0.02–4.72)	0.69	2.73(0.48–15.36)	0.26	4.41(1.25–15.62)	0.02
SHS	0.08(0.002–2.34)	0.14	0.72(0.16–3.32)	0.67	1.92(0.72–5.16)	0.19
Tertiary	-	-	0.69(0.07–6.35)	0.74	2.21(0.53–9.25)	0.28
**Birthweight of last pregnancy**						
Normal **(ref.)**	-	-	1		1	
Overweight	-	-	1.52(0.0633.72)	0.79	3.13(0.35–28.32)	0.31
Underweight	-	-	1.72(0.08–36.15)	0.73	3.24(0.31–33.79)	0.32
**Total cholesterol**						
Normal	1		1		1	
High	1.78(0.58–5.48)	0.07	3.60(0.43–29.92)	0.23	0.81(0.26–5.51)	0.72
**Triglyceride**						
Normal	-	-	1		1	
High	-	-	0.63(0.11–3.60)	0.60	0.35(0.09–1.28)	0.11
**HDL**						
Normal	1		1		1	
Low	0.76(0.03–17.18)	0.87	1.11(0.18–6.67)	0.90	2.00(0.56–7.14)	0.28
**LDL**						
Normal	1		1		1	
High	6.9(0.36–13.14)	0.11	1.11(0.29–4.24)	0.87	0.74(0.29–1.87)	0.52

HDL: high-density lipoprotein cholesterol; LDL: low-density lipoprotein cholesterol

## Discussion

The current study involved 252 pregnant women who were attending the ANC at the CCTH. A 0.8% and 19.44% prevalence of GDM and GGI respectively were found in the current study. In the entire sample without any sub-group analysis, JHS education increased the odds but being in the 2^nd^ trimester decreased the odds of having insulin resistance. In a trimester-based sub-group analysis, being in the 3^rd^ trimester with JHS education increased the odds of having insulin resistance.

Pregnancy is a normal physiological process necessary for procreation. It is associated with insulin resistance [24–26]. Although insulin resistance can be advantageous to the foetus because it directs glucose towards foetal growth [25–27], it can also have adverse effects if uncontrolled. Indeed, insulin resistance is associated with GDM [28] known to be linked to adverse pregnancy outcomes [29]. The GDM prevalence of 0.8% observed in the current study is lower than those of other studies. For instance, studies in Kumasi [30] and Accra [3, 31], have reported GDM prevalence of 8.5% and 9.3–10% respectively. Others have reported 11.6–19.8% in India [21, 32], 13.61-14% for the African continent [18, 33] and 35% in Bangladesh [34]. The huge disparity in the observed prevalence of GDM between the current study and the previous ones [3, 18, 30, 31, 33, 34] could be ascribed to probable differences in study design and characteristics of participants. In the current study, pregnant women who had been previously diagnosed of diabetes of any kind were excluded from the study unlike the previous reports that included such participants. In addition, participants in the current study were generally younger than those of the previous studies [3, 30]. Indeed, maternal age above 35 years is a known risk factor for development of GDM. Thus, the lower prevalence of GDM could be a reflection of lower number of risk factors for GDM among participants in the current study as opposed to those of the previous studies that reported higher prevalence values. Our results also point to the need for contextualisation in application of prevalence data for decision-making.

However, the diagnosis of GDM in the current study fell within the 3^rd^ trimester, in line with the recommended period of gestation for diagnosing the condition. As pregnancy progresses, levels of human placental lactogen, corticotrophin-releasing hormone, progesterone, human placental growth hormone, estrogen, prolactin, adiponectin, leptin, resistin, interleukin-6 and tumour necrosis factor alpha are altered in circulation [35, 36] and impair insulin signalling resulting in reduced glucose uptake in spite of increased insulin level and, thus promote insulin resistance. Moreover, increased maternal adiposity in early pregnancy is thought to promote free fatty acid release in late pregnancy, which impairs glucose uptake and promotes hepatic gluconeogenesis to further worsen the pregnancy-associated insulin resistance [24, 37]. The above considerations are thought to underpin the recommendation to test for GDM in middle to late pregnancy where insulin resistance is expected to have peaked [27, 36]. As an adaptation mechanism to pregnancy, the beta-cell insulin secretory function increases to accommodate the imposed increased demand [38] suggesting that the presence of insulin resistance in pregnancy is not a problem but the body’s ability to respond appropriately in a sustainable manner to assuage its probable adverse effect of clinical significance, is the challenge. In the current study, neither insulin resistance nor beta-cell secretory function, as assessed by the homeostatic model [22], showed a statistical difference across the trimesters of pregnancy. This observation could be due probably to the relatively small sample size per trimester of pregnancy. However, overall adiposity in terms of BMI increased across the trimesters of pregnancy, in support of established norms [3, 18, 27, 30, 31, 33, 34, 36]. Moreover, the observed 56.75% prevalence of insulin resistance in the current study does not necessarily indicate any risk of future development of type 2 diabetes mellitus (T2DM) as long as the adaptive mechanisms in pregnancy are effective enough to regulate blood glucose within the acceptable limit.

Interestingly, a 19.44% prevalence of GGI was found suggesting a reduced effectiveness of the expected adaptive mechanisms to minimise the impact of insulin resistance in our setting. Although GGI may easily be overlooked, a recent retrospective cohort study [39] involving 16,836 individuals who were followed for prenatal and primary care over a median period of 8.4 years in the USA, has demonstrated a higher diabetogenic risk of the phenomenon compared with those with normal glucose tolerance. This suggests that persons with GGI must be considered a high-risk group, just like those with GDM, and closely monitored for appropriated preventive management, against probable future development of T2DM. In the Ghanaian context, this is the first report of GGI, to the best of our knowledge. Our GGI prevalence rate of 19.44% is higher than a 16.8% reported by Gautam et al. [21], suggesting a higher burden and in furtherance of the urgent need to take appropriate preventive measures to minimise the probable effect. The higher prevalence of GGI in our setting compared to the Indian study [21] could be ascribed to differences in characteristics of participants. Participants for the Indian study [21] were younger with a mean age of 25 years compared with the current one with mean age 30 years.

In malaria-endemic countries like Ghana, the effects of malaria on insulin resistance and pregnancy outcomes cannot be overemphasized. A number of previous studies have demonstrated the capacity of infection by the malaria parasite to induce insulin resistance in human [6, 7] and rat [40] studies. Malaria-associated insulin resistance results from interaction of adipokines such as leptin and adiponectin with markers of inflammation and oxidative stress [41, 42] in non-pregnant individuals similar to that observed for pregnancy-related insulin resistance [35, 36]. With malaria remaining the leading cause of morbidity in Ghanaian health facilities [9] coupled with its negative impact on pregnancy outcomes if untreated, the government of Ghana has long adopted a WHO recommendation that allows malaria to be treated prophylactically for all pregnant women as part of the routine antenatal care with sulfadoxine–pyrimethamine [15]. Interestingly, malaria could not be detected in any of the study participants, indicating that the implementation of the IPTp–SP recommendation together with other malaria control programmes [9] have been highly effective in our setting in support of earlier reports [15, 43] since all participants had been treated. Although microscopy remains the gold standard for malaria diagnosis, a recent Ghanaian study [44] has affirmed the superiority of rapid diagnostic test (RDT). Indeed, the accuracy of microscopy is operator-dependent suggesting that a combination with RDT may be more useful, especially in instances of low parasite levels.

Our logistic regression analyses showed that, participants with JHS education demonstrated a 2.53 increased odds of having insulin resistance compared with those without formal education in the entire sample. A sub-group analysis revealed an increased odds of 4.41 for those in the 3^rd^ trimester with JHS education compared with their counterparts without any formal education and in the 1^st^ trimester of pregnancy after controlling for confounders. Whereas the increased odds of insulin resistance in the 3^rd^ trimester supports the established physiological pattern in pregnancy [18, 26, 29, 33, 35], that of the JHS level of educational attainment requires further investigation to ascertain the biochemical and clinical significance. Moreover, the observed 68% reduction of odds of having IR in the 2^nd^ trimester compared to the 1^st^ trimester of pregnancy requires further investigation for possible confirmation and elucidation of biochemical mechanisms as well as clinical significance because it deviates from the expected pattern [18, 26, 29, 33, 35].

The current study has several limitations that ought to be taken into consideration in applying the findings to other populations within the appropriate context. First, this is a cross-sectional study, making establishment of causality challenging. Also, the confinement of the study to only a tertiary healthcare facility in the Central region of Ghana coupled with the relatively small sample size, calls for caution in undue extrapolation of findings to populations of widely different characteristics to those of the current study. Above all, other lifestyle variables such as exercise, nutritional and dietary factors as well as other pregnancy-associated hormones that are known to contribute to the development of insulin resistance could not be assessed in the current study. Therefore, a longitudinal study involving a larger sample size with a wider coverage that assesses detailed lifestyle and biochemical indices may be necessary to confirm the current findings to guide policy decisions. Notwithstanding the above limitations, the current report has provided evidence of existence of GGI that must be given the needed attention in our quest to minimise the diabetogenic risk associated with pregnancy and post-partum in our setting.

## Conclusion

Prevalence of GDM was 0.8% with that of GGI and insulin resistance being 19.44% and 56.75% respectively. Being in the 3^rd^ trimester with JHS education increased the odds of having insulin resistance in our setting. Appropriate measures are needed to assuage the diabetogenic risk posed by GGI in our setting.

### Electronic supplementary material

Below is the link to the electronic supplementary material.

Supplementary Material 1

## Data Availability

The questionnaire used has been attached as supplementary information. Every data is provided within the manuscript.

## Abbreviations

ANC: Antenatal clinic
BMI: Body mass index
CCTH: Cape Coast Teaching Hospital
FPS: Fasting plasma glucose
GDM: Gestational diabetes mellitus
GGI: Gestational glucose intolerance
HDL: High-density lipoprotein cholesterol
HOMA-B: Homeostatic model assessment of beta-cell function
HOMA-IR: Homeostatic model assessment of insulin resistance
IDF: International Diabetes Federation
IPTp–SP: Intermittent preventive treatment in pregnancy with sulfadoxine– pyrimethamine
IR: Insulin resistance
LDL: Low-density lipoprotein cholesterol
OGTT: Oral glucose tolerance test
RDT: Rapid diagnostic test
TC: Total cholesterol
WHO: World Health Organization
