# Supplementary material for: Gestational glucose intolerance among pregnant women at the Cape Coast Teaching Hospital
Source: BMC Pregnancy Childbirth. 2024 May 14;24:356. doi: 10.1186/s12884-024-06568-y (PMC11092027; doi:10.1186/s12884-024-06568-y)
Supplement: Supplementary file 1 — Supplementary Material 1 [file 12884_2024_6568_MOESM1_ESM.docx]

## Supplementary 1: Study Questionnaire

Please tick (√) or fill where appropriate.

A. **SOCIO DEMOGRAPHIC DATA**

**PARTICIPANTS ID:………………………………**

**AGE……………………………..**

**RESIDENCE:……………………………………………………………………..**

**1.**How old is the pregnancy**:** □**1wk-12wk** □13wk-24wk □25wk-36wk

2.Weight Height: …………………. BMI

**3.** Do you sleep in mosquito treated bed net: Yes□ No□

**4.**If yes, how often: Sometimes□ Always□

**PAST OR CURRENT MEDICAL PROBLEMS**

| Have you ever been diagnosed of any of the underlisted conditions?  Diabetes  High blood pressure  heart disease  Gestational Diabetes  Obesity  Polycystic Ovary Syndrome  Malaria | Yes  □  □ □ □ □ □ | No  □ □ □ □ □ □ |
| --- | --- | --- |

**B. SOCIAL HISTORY**5.Have you ever smoked? Yes□ No □ Current smoker q Quit (month/year):

6.If yes, how many packs per day? □<1 □ 1 □2 □ >3 For how many years?

7.Do you drink alcohol? □ Yes □No

If yes, how many drinks per week? □<1 □1–4 □ 5–10 □ >20

8.What is your occupation?

Health Professional □ Teacher □ Lawyer □ House wife □ Trader□ Other…………………….

9.Marital status: □ Single □Partnered/Married □ Divorced □ Widowed □ Other

10.Highest level of education: □ Elementary □ Junior High □ Senior High Tertiary

**C.OBSTETRIC HISTORY**

11.Is this your first Pregnancy Yes□ No □

If No how many:…………………………………

12.Did the pregnancy get to full term Yes□ No □

13.Was it a normal delivery Yes□ No □

14.Was it by caesarean section Yes□ No □

15.Was the baby alive Yes□ No □

16.Was there any difficulty during delivery Yes□ No □

17.If yes can you share with us the difficulty………………………………………………….

18.What was the weight of the baby: □ 2.5Kg □≥2.5Kg Others Specify…………….…..
